# Evaluating the Quality of Systematic Reviews and Meta-Analyses About Breast Augmentation Using AMSTAR

**DOI:** 10.1093/asjof/ojab020

**Published:** 2021-05-22

**Authors:** Morgan Yuan, Jeremy Wu, Ryan E Austin, Frank Lista, Jamil Ahmad

**Affiliations:** Michael G. DeGroote School of Medicine, McMaster University, Hamilton, ON, Canada; University of Toronto, Toronto, ON, Canada; Division of Plastic, Reconstructive & Aesthetic Surgery, Department of Surgery, University of Toronto, Toronto, ON, Canada; Division of Plastic, Reconstructive & Aesthetic Surgery, Department of Surgery, University of Toronto, Toronto, ON, Canada

## Abstract

**Background:**

Breast augmentation is one of the most commonly performed cosmetic surgeries worldwide. Therefore, it is imperative to have evidence with high methodological quality to guide clinical decision making.

**Objectives:**

To evaluate the methodological quality of the systematic reviews (SRs) focused on breast augmentation.

**Methods:**

A comprehensive search of MEDLINE, Embase, and the Cochrane Library of Systematic Reviews was performed. SRs that have a particular focus on breast augmentation and were published in the top 15 plastic and reconstructive surgery journals were included. Quality assessment was performed using a measurement tool to assess systematic reviews (AMSTAR). Study characteristics were extracted including journal and impact factor, year of publication, country affiliation of the corresponding author, reporting adherence to Preferred Reporting Items for Systematic Reviews and Meta-Analyses (PRISMA) guidelines, number of citations, and number of studies included.

**Results:**

Among the 22 studies included for analysis, the mean AMSTAR score was moderate (5.55), with no SR achieving good quality (AMSTAR score of ≥9). There were no significant associations between AMSTAR score and journal impact factor, number of citations, year of publication, or number of included studies. Studies that reported adherence to PRISMA guidelines on average scored higher on the AMSTAR tool (*P* = 0.03).

**Conclusions:**

The methodological quality of reviews about breast augmentation was found to be moderate, with no significant increase in studies or quality over time. Adherence to PRISMA guidelines and increased appraisal of SRs about breast augmentation using methodological assessment tools would further strengthen methodological quality and confidence in study findings.

Breast augmentation is one of the most commonly performed cosmetic surgeries around the world.^1-3^ While in concept breast augmentation is a straightforward procedure (ie, the placement of a breast implant to provide an improved aesthetic outcome), there are actually many factors that a plastic surgeon must consider when performing breast augmentation, including implant type, pocket selection, breast anatomy, and patient aesthetic preferences.^1-3^ Though women report increased satisfaction with breast self-image, psychosocial well-being, sexual functioning, and self-confidence, these surgeries are associated with potential complications including capsular contracture, implant failure, and, uncommonly, breast implant-associated anaplastic large cell lymphomas (BIA-ALCL).^2^ Therefore, plastic surgeons performing breast augmentation require evidence-based recommendations with strong methodological quality to help guide these clinical decisions.

With the rapidly growing body of medical literature, clinicians must remain up-to-date with the latest research to provide the highest quality treatment. Rather than sorting through dozens of original articles, it is more efficient to consult systematic reviews or meta-analyses that compile and summarize the existing literature.^3^ Through an objective and methodological approach in assessing literature, systematic reviews and meta-analyses can extract and synthesize information to draw conclusions from the current pool of evidence. However, these studies vary in quality and have been found to show disparities when tackling the same research question, which brings the reliability of these types of studies into question. Fortunately, tools have been developed to guide and assess the quality of systematic reviews and meta-analyses. The Preferred Reporting Items for Systematic Reviews and Meta-Analyses (PRISMA) statement is a 27-item checklist for improving transparent reporting of systematic reviews and meta-analyses.^4^ This checklist allows studies to be critically examined for their strengths and weaknesses. Similarly, Shea et al developed a measurement tool to assess systematic reviews (AMSTAR) criteria to assess the methodological quality of systematic reviews by building upon preexisting instruments, coupled with empirical evidence and expert consensus.^5^ This 11-item tool can be used to identify the quality of design in systematic reviews and meta-analyses, and subsequently, the strength of conclusions drawn. This tool has been used in different specialties including urology^6,7^ and hand surgery in the domain of plastic surgery.^8^ In conducting this present study, we adhered to the PRISMA and AMSTAR criteria when applicable.

Considering the popularity of breast augmentations, the quality of systematic reviews and meta-analyses that guides this procedure need to be conducted using thorough and valid methods. To the best of our knowledge, no previous studies have assessed the quality of systematic reviews and meta-analyses in breast augmentation. Therefore, the primary objective of this study was to evaluate the methodological quality of systematic reviews and meta-analyses in breast augmentation surgery that have been published in major plastic and reconstructive surgery journals. The secondary objective was to discern whether study characteristics (eg, number of citations, impact factor of journal, year of publication, adherence to PRISMA guidelines) were associated with the quality of the systematic reviews and meta-analyses.

## METHODS

### Search Strategy

A comprehensive literature search of MEDLINE, Embase, and the Cochrane Library of Systematic Reviews was performed in June 2020 to identify all systematic reviews published between January 2000 and December 2019 in the top 15 plastic and reconstructive surgery journals as ranked by impact factor (Table 1). The search strategies used for each database are available in the Appendix (available as Supplemental Material at www.asjopenforum.com).

**Table 1.** Top 15 Plastic and Reconstructive Surgery Journals by 2019 Web of Science Impact Factor

Journal title	Impact factor
*Plastic and Reconstructive Surgery*	4.235
*Aesthetic Surgery Journal*	3.799
*JAMA Facial Plastic Surgery*	3.787
*Journal of Plastic, Reconstructive and Aesthetic Surgery*	2.390
*Journal of Hand Surgery (European Volume)*	2.290
*Journal of Hand Surgery (American Volume)*	2.124
*Burns*	2.066
*Microsurgery*	1.996
*Clinics in Plastic Surgery*	1.959
*Journal of Reconstructive Microsurgery*	1.841
*Aesthetic Plastic Surgery*	1.798
*Journal of Cranio-maxillofacial Surgery*	1.766
*Journal of Burn Care & Research*	1.533
*Annals of Plastic Surgery*	1.354
*Facial Plastic Surgery*	1.108

The studies identified by the search were uploaded to Covidence software for systematic reviews (Veritas Health Innovation Ltd, Melbourne, Australia). Articles with duplicate titles were removed. Two authors (M.Y. and J.W.) independently screened article titles and abstracts for inclusion in the subsequent analysis. Any studies where the information presented in the title and abstract was insufficient to determine eligibility were reviewed by full-text screening. All discrepancies throughout the 2-stage screening process were resolved through consensus.

### Eligibility Criteria

Only articles with a particular focus on breast augmentation, which were identified as systematic reviews or meta-analyses in the title and/or text, or reviews that specifically indicated a systematic search strategy to identify studies were included for analysis. Studies that were non-English literature, nonhuman-based studies, systematic reviews of systematic reviews, and other study designs (case studies, narrative reviews, expert opinions, editorials, protocols, and conference abstracts) were excluded.

### Data Collection and Analysis

Data from the included reviews were extracted independently by 2 authors. Any discrepancies were resolved by discussion and consensus. In addition to quality assessment using the AMSTAR tool, parameters from included studies were extracted, including journal impact factor in 2019 (Web of Science, Clarivate Analytics, Philadelphia, PA), year of publication, country of origin based on the corresponding author, reporting adherence to PRISMA guidelines, number of Google Scholar citations (collected on June 10, 2020), and number of studies included. Study findings and conclusions were collected and synthesized based on the interventions compared and the outcomes assessed for these interventions.

### Quality Assessment

The AMSTAR tool was used to assess the methodological quality of studies (Table 2). The 11-item measurement tool assigns a score of 0 or 1 for each criterion, with total scores ranging from 0 to 11. AMSTAR scores of 4 or less are classified as “poor methodological quality,” scores of 5-8 as “moderate methodological quality,” and scores of 9 or greater as “good methodological quality.” Two review authors independently selected “yes,” “no,” or “not applicable” for each criterion. Any discrepancies were resolved through consensus. One point was given to each criterion that received a “yes,” while no points were awarded for “no” and “not applicable.”

**Table 2.** Assessment of Multiple Systematic Reviews Criteria

AMSTAR criteria	Description
1	An “a priori” design was provided
2	Duplicate study selection and data extraction
3	Comprehensive literature search
4	Status of publication used as inclusion criteria
5	List of studies provided
6	Characteristics of included studies provided
7	Scientific quality of included studies provided
8	Scientific quality of included studies used appropriately in formulating conclusions
9	Appropriate methods used to combine findings of studies
10	Likelihood of publication bias assessed
11	Conflict of interest stated

AMSTAR, a measurement tool to assess systematic reviews.

Microsoft Excel (Microsoft Corporation, Redmond, WA) was used to construct tables and graphs to summarize the results. Statistical analysis was performed using GraphPad Prism software (version 7.0, GraphPad Software, Inc, San Diego, CA). Pairwise correlations (AMSTAR score as compared with citation number, impact factor, year of publication, number of studies included) were evaluated using the Pearson correlation coefficient (*r*). The difference in AMSTAR score by adherence to PRISMA guidelines was evaluated with a 2-tailed *t* test. For this study, *P* values of less than 0.05 were considered statistically significant.

Cohen’s kappa (κ) statistic was used to assess the interrater reliability, with values of 0.01-0.20 (“slight agreement”), 0.21-0.40 (“fair agreement”), 0.41-0.60 (“moderate agreement”), 0.61-0.80 (“substantial agreement”), and 0.81-0.99 (“almost perfect agreement”), respectively.^9^

## RESULTS

### Search Results

**Figure 1.**
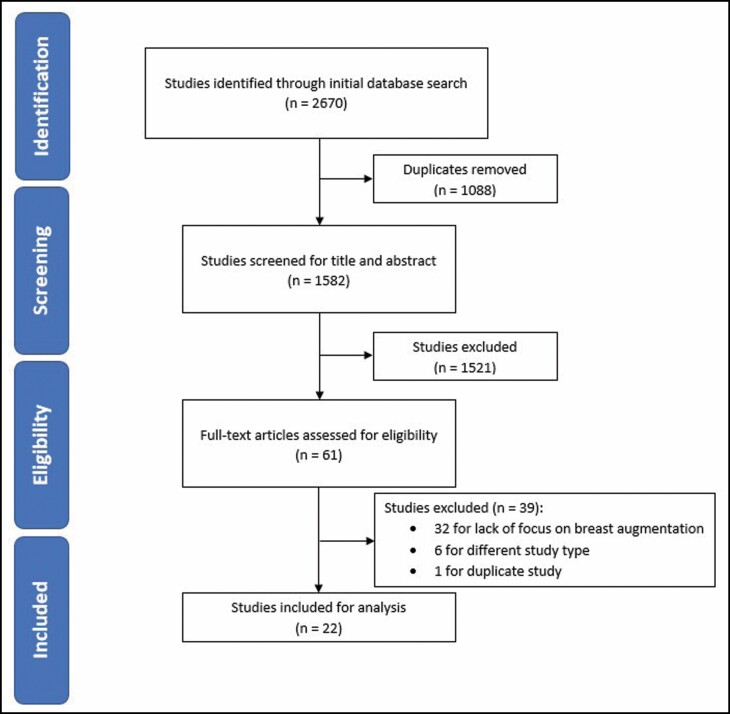
PRISMA diagram demonstrating results of literature search. PRISMA, Preferred Reporting Items for Systematic Reviews and Meta-Analyses.

The initial search identified 2670 studies, with 1088 duplicates (41%) removed (Figure 1). The remaining 1582 studies proceeded to abstract/title screening with 1521 (57%) of these being deemed ineligible. This left 61 studies (2.3%) that moved onto full-text screening. Amongst these 61 studies, 39 studies (1.5%) were excluded based on a lack of focus on breast augmentation (32), not being a systematic review or meta-analysis (6), and for being a duplicate (1). After completing both stages of screening, 22 (0.82%) studies were included for analysis.^10-31^ Cohen’s kappa was found to be 0.814 at title/abstract screening, which indicates almost perfect agreement between the 2 reviewers and strong interrater reliability.

### General Study Characteristics

**Figure 2.**
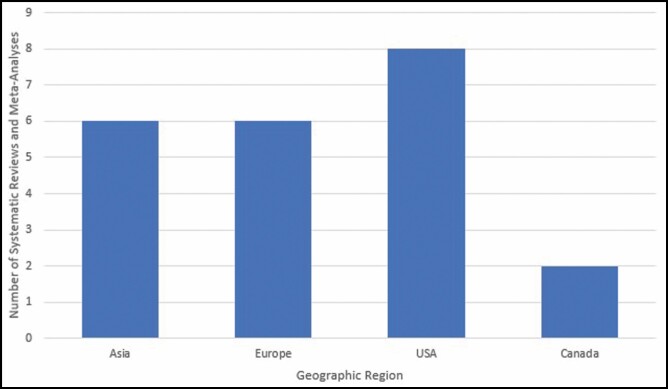
Number of systematic reviews and meta-analyses included by geographic region of the corresponding author.

**Figure 3.**
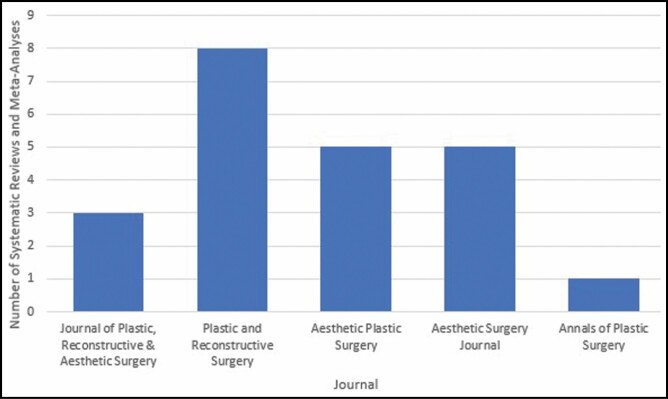
Number of systematic reviews and meta-analyses by journal.

Study characteristics are summarized in Table 3. About 36% of articles were published by authors from the United States (n = 8), with the second most from authors in China (n = 4, 18%) (Figure 2). Similarly, 36% of articles were published in *Plastic and Reconstructive Surgery* (n = 8), followed by 23% in both *Aesthetic Plastic Surgery* (n = 5) and the *Aesthetic Surgery Journal* (n = 5) (Figure 3). At least 1 study was published each year between 2010 and 2019, with 5 (23%) studies published in 2015. The number of studies included in each systematic review/meta-analysis ranged from 5 to 42, with an average of 18.5 studies included in each study. The average number of citations per study was 52.1, with the maximum number of citations being 294. Nine (41%) studies adhered to PRISMA guidelines while 13 (59%) did not.

**Table 3.** Characteristics of Included Studies

Author	Journal	Year	Country affiliation	Google Scholar citations	No. of studies	PRISMA adherence
Shen	*Journal of Plastic, Reconstructive & Aesthetic Surgery*	2019	China	3	19	1
Khavanin	*Plastic and Reconstructive Surgery*	2014	United States	61	23	0
Larcher	*Aesthetic Plastic Surgery*	2015	Austria	15	7	0
Voglimacci	*Aesthetic Surgery Journal*	2015	France	27	42	1
Rosing	*Aesthetic Plastic Surgery*	2011	United States	97	17	0
Groen	*Aesthetic Surgery Journal*	2016	Netherlands	36	22	1
Noels	*Aesthetic Surgery Journal*	2015	Netherlands	24	17	1
Lynch	*Aesthetic Plastic Surgery*	2018	United States	17	7	1
Wong	*Plastic and Reconstructive Surgery*	2006	Singapore	280	6	0
Li	*Aesthetic Plastic Surgery*	2017	China	17	7	0
Schaub	*Plastic and Reconstructive Surgery*	2010	United States	75	16	0
Li	*Aesthetic Plastic Surgery*	2019	China	5	11	0
Yalanis	*Plastic and Reconstructive Surgery*	2015	Taiwan	53	9	1
Largo	*Journal of Plastic, Reconstructive & Aesthetic Surgery*	2013	Germany	146	36	1
Drinane	*Annals of Plastic Surgery*	2017	United States	16	8	1
Adams	*Plastic and Reconstructive Surgery*	2016	Canada	20	33	0
Ducic	*Aesthetic Surgery Journal*	2014	United States	24	36	0
Stanley	*Aesthetic Surgery Journal*	2012	United States	29	12	0
Wan	*Plastic and Reconstructive Surgery*	2015	United States	78	25	0
Cheng	*Plastic and Reconstructive Surgery*	2018	China	7	5	0
di Summa	*Journal of Plastic, Reconstructive & Aesthetic Surgery*	2018	Switzerland	7	41	1
Barnsley	*Plastic and Reconstructive Surgery*	2005	Canada	332	7	0

PRISMA, Preferred Reporting Items for Systematic Reviews and Meta-Analyses.

The conclusions of the included studies were classified based on the interventions assessed and their measured outcomes. The predominant interventions among these studies were general augmentation mammaplasty, autologous fat grafting, implant-based augmentation, augmentation-mastopexy, and surgical site irrigation. The conclusions derived from these studies were classified as relating to complications, patient-reported outcome measures, objective outcomes, and other conclusions. These conclusions and the recommendations offered have been collated into Table 4.

**Table 4.** Summary and Synthesis of Conclusions Identified Within Our Included Studies

Intervention assessed	Complications	Patient-reported outcome measures	Objective outcomes	Other conclusions
General augmentation mammaplasty	- Periareolar incisions showed higher rates of CC formation compared with inframammary and transaxillary incisions^19^ - There is a possibility of nerve injury, sensation change, and chronic pain after augmentation-mammaplasty, which requires timely treatment to optimize patient outcomes^26^ - Acellular dermal matrices and site changes are recommended for CC management after augmentation procedures due to low CC recurrence rates^28^	- There is an overall high patient and surgical team satisfaction^10^	- No objective outcomes of general augmentation procedures were assessed among our included studies	- Postoperative pain relief requiring responsibility from the patient is not preferred, as they are not more efficacious than those administered by the healthcare team^27^ - Selection of postoperative pain relief should be based on cost and ease of implementation for both the patient and surgeon^27^
Autologous fat grafting	- Low overall complication rate (10%-20%), comparable to implant-based augmentation^15,23^ - Most prevalent complications in AFG are benign calcifications, palpable indurations, and surgical site infections^13-15^	- High levels of patient satisfaction following AFG^13,15,23^	- Insufficient data to conclude long-term safety of AFG, but AFG appears to be associated with a low chance of developing breast cancer^13-15,23^ - AFG shows some degree of lasting improvement in breast size and shape^14,23^ - If long-term safety and good volume gain is identified, AFG should be recommended to patients interested in breast augmentation^23^	- No evidence-based preferences for fat harvesting, or processing^13,14^ - Most clinicians prefer to avoid fat reinjection in any glandular tissue, but no preference between submammarily, subcutaneously, or intrapectorally injections^13,14^ - AFG should be reserved for trained and highly skilled teams and must follow strict recommendations due to lack of established evidence^13^
Implant-based augmentation	- Textured implants are favored over smooth implants due to lower CC formation that persists at 3 years follow-up; no other complications were found to be significantly different between these implant types^18,31^ - Implants in submuscular plane have decreased CC rates^20^ - Implant exchange can be used as a management option for CC^18^ - Subpectoral placement associated with fewer complications (CCs, hematomas, and seromas) than subglandular and subfascial^10^ - Subpectoral associated with lower rates of CC and hematomas than prepectoral, but higher implant displacement and animation deformity^21^	- Perception of breast hardness is not significantly different between textured and smooth implants, but smooth implants are preferred due to less palpability^18,20^ - High satisfaction rates with implants, regardless of implant plane, based on patient surveys^10,20^ - Both round and anatomical implants achieved similar aesthetic effects, but round implants are recommended due to unique risks associated with anatomical implants^29^	- Implant-based augmentation is associated with lower rates of breast cancer than the general population^16^	- Decision on type of implant used should be guided by surgeon’s personal experience^20^ - Due to significant heterogeneity in the literature, more rigorous quantitative methods are required to assess implant size selection systems^25^
Augmentation-mastopexy	- Pooled complication and reoperation rates are comparable to published rates for primary augmentation or mastopexy alone^11^ - Management of postsurgical pyoderma gangrenosum following augmentation-mastopexy requires limited surgical interventions until controlled with corticosteroids^12^	- Mastopexies are generally associated with high satisfaction rates^30^	- There is a low incidence of insufficient breast life, especially seen in mastopexy with glandular reshape^30^	- Augmentation-mastopexy requires careful patient selection to be safe and effective^11^ - Prophylactic antibiotics should be added to prevent bacterial infections in high-risk patients following augmentation-mastopexy^12^
Surgical site irrigation	- No association between povidone-iodine irrigation and implant deflation and rupture^22^ - Conflicting evidence on rate of CC formation with povidone-iodine/antimicrobial irrigation^17,22,24^	- Best method of non-narcotic pain relief post-augmentation involves pocket irrigation with bupivacaine and ketorolac due to its simplicity, efficacy, and administration intraoperatively that removes responsibility from the patient^27^	- No objective outcomes on surgical site irrigation were assessed among our included studies	N/A

AFG, autologous fat grafting; CC, capsular contracture.

### Overall Methodological Quality of Included Studies

**Figure 4.**
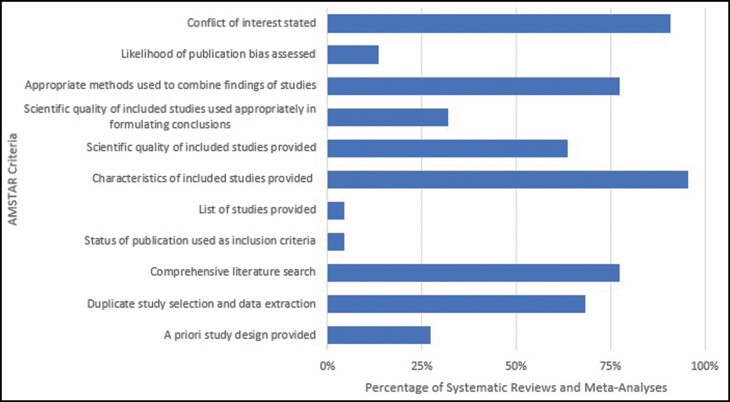
Percentage of systematic reviews and meta-analyses adhering to each AMSTAR criteria. AMSTAR, a measurement tool to assess systematic reviews.

The overall methodological quality, as measured by AMSTAR score (/11), varied between 2 and 8, with no studies achieving a “good quality” AMSTAR score of ≥9. The average AMSTAR score was moderate (5.55 ± 1.65). Adherence to specific AMSTAR criteria was inconsistent across the studies (Figure 4). AMSTAR criteria 6 and 10 had the greatest adherence, with 21/22 (95%) studies providing characteristics of included studies and 20/22 (91%) studies stating any conflicts of interest. Conversely, the AMSTAR criteria with the lowest adherence were criteria 4 and 5, with 1/22 (4.5%) studies including gray literature and providing a list of excluded studies after screening.

### Factors Associated With Methodological Quality

**Figure 5.**
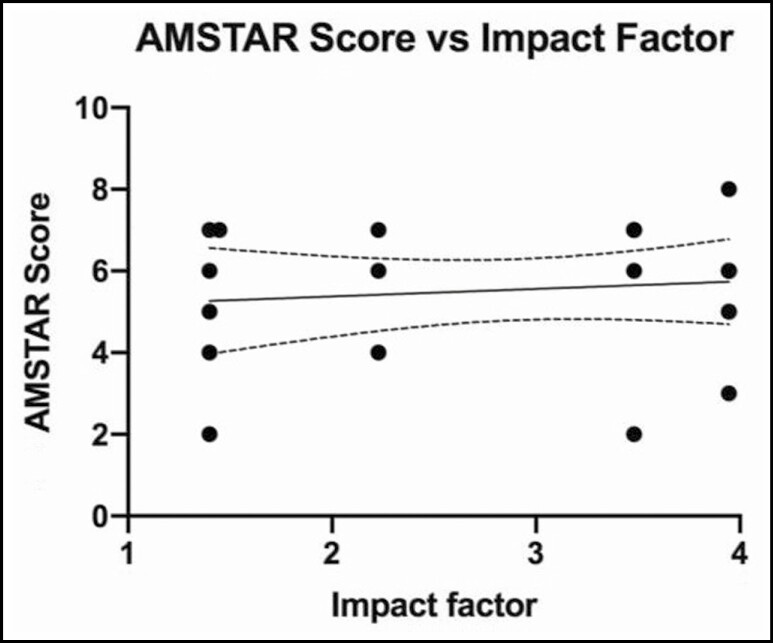
AMSTAR score as compared with journal impact factor. AMSTAR, a measurement tool to assess systematic reviews.

**Figure 6.**
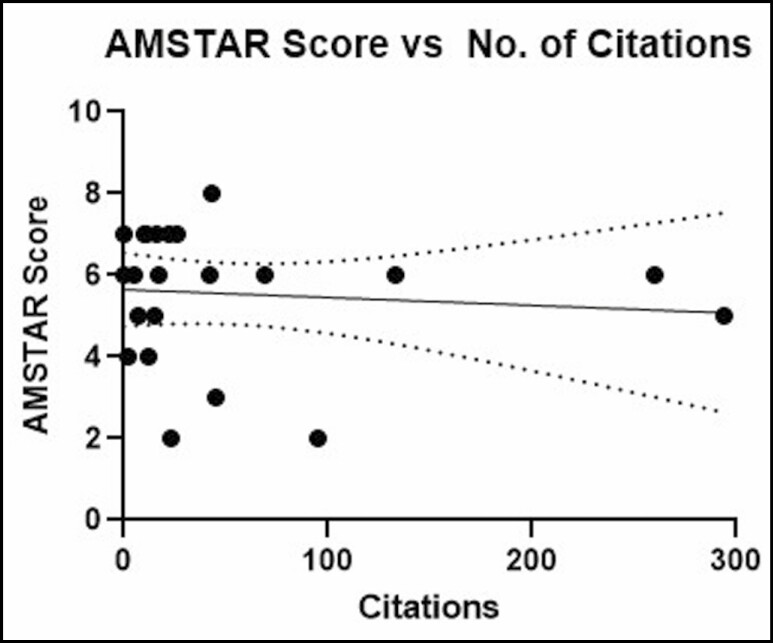
AMSTAR score as compared with number of Google Scholar citations. AMSTAR, a measurement tool to assess systematic reviews.

**Figure 7.**
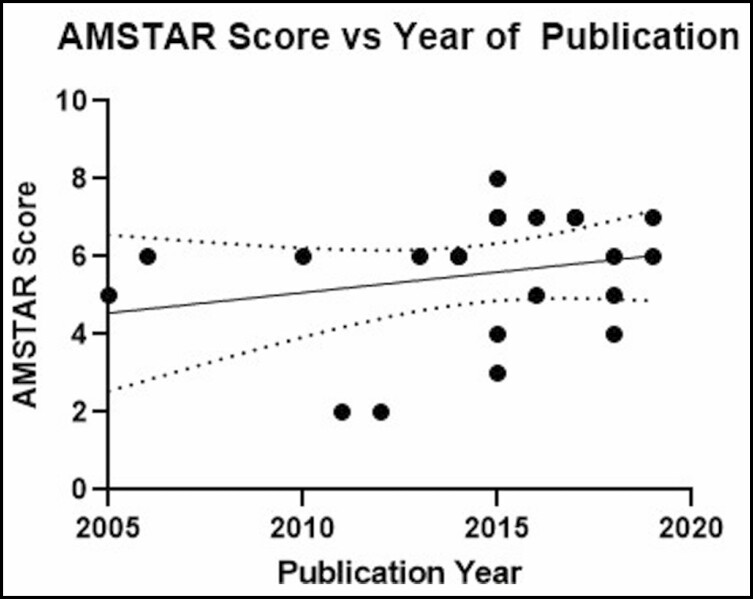
AMSTAR score as compared with year of publication. AMSTAR, a measurement tool to assess systematic reviews.

**Figure 8.**
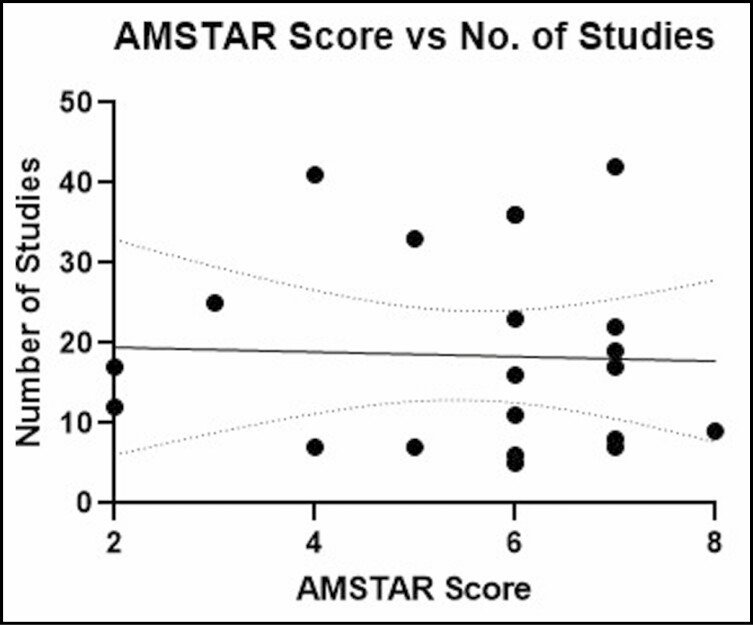
AMSTAR score as compared with number of included studies. AMSTAR, a measurement tool to assess systematic reviews.

**Figure 9.**
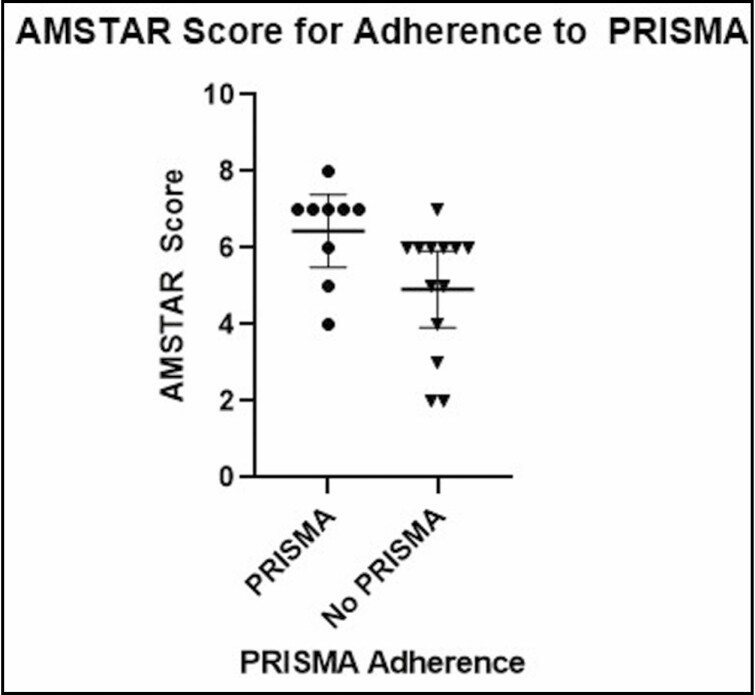
AMSTAR score as compared with PRISMA adherence. AMSTAR, a measurement tool to assess systematic reviews; PRISMA, Preferred Reporting Items for Systematic Reviews and Meta-Analyses.

No significant relationships were found between mean AMSTAR score and journal impact factor (*P* = 0.5883; *r* = 0.1221; 95% CI = −0.32, 0.52; Figure 5), number of citations (*P* = 0.6802; *r* = 0.0931; 95% CI = −0.012, 0.0077; Figure 6), year of publication (*P* = 0.2810; *r* = 0.2405; 95% CI = −0.093, 0.30; Figure 7), and number of included studies (*P* = 0.8640; *r* = 0.0388; 95% CI = −3.73, 3.12; Figure 8). Studies that adhered to PRISMA guidelines had a higher AMSTAR score on average compared with those that were not PRISMA adherent (*P* = 0.03) (Figure 9).

## DISCUSSION

With the rapidly growing body of literature, clinicians can remain up-to-date with the latest research by consulting systematic reviews and meta-analyses that summarize the existing evidence.^3^ However, these studies have been found to show disparities when tackling the same research question, which is problematic as clinicians often apply the conclusions from these studies into clinical care.^3^ Fortunately, the AMSTAR tool has been developed to assess the methodological quality of systematic reviews by identifying the quality of the study design and the strength of the conclusions drawn.^4^ Given the popularity of breast augmentation procedures and the potential complications associated with them, a quality assessment of systematic reviews and meta-analyses that guide this procedure needs to be conducted to ensure that these studies are performed using thorough and valid methods.^1,2^ Therefore, the primary goal of this study was to apply the AMSTAR criteria to systematic reviews and meta-analyses in breast augmentation surgery that are published in major plastic and reconstructive surgery journals. The secondary goal of this study was to discern whether study characteristics were associated with the AMSTAR score of systematic reviews and meta-analyses.

According to a review by Oxman et al, AMSTAR was considered the best criteria for appraising systematic reviews among the available options.^32^ Despite the fact that AMSTAR was not developed for evaluating systematic reviews of non-randomized studies, Pieper et al showed that AMSTAR has good psychometric properties when used in these studies.^33^ Within our included studies, many were conducted on non-randomized trials. As such, the AMSTAR tool was applied to our final list of systematic reviews and meta-analyses.

Previously, several studies have assessed the methodological quality of systematic reviews within the domain of plastic surgery. Samargandi et al identified that across systematic reviews in the journal of *Plastic and Reconstructive Surgery*, there was an increase in the number of systematic reviews, but not the methodological quality over time.^34^ They indicated that peer-review processes in *Plastic and Reconstructive Surgery*, which has the highest impact factor among plastic surgery journals, was not adequate and that articles should also be reviewed by experts in epidemiological methods. A recent study by McGuire et al found that there was an increase in frequency and quality of meta-analyses in plastic surgery.^35^ However, they found that overall evidence was still low. Momeni et al found that within the subdomain of hand surgery, the number of systematic reviews and methodological quality increased over time.^36^ Our findings may differ from this study since Momeni et al felt only a list of included studies met AMSTAR criteria 5, while the present study required a list of excluded studies as well to meet the criterion. However, this study concluded that further improvement in methodological quality is needed despite the quality improvement over time.

From our analysis, we did not find any significant change in the number of studies or methodological quality over time. We reported an average AMSTAR score of 5.55 in our included studies, indicating an overall moderate quality of studies published about breast augmentation. Systematic reviews are often regarded as the highest level of evidence and are used to guide clinical practice. If the systematic reviews on breast augmentation are only able to score, on average, 5.55 points of the total 11, this means that the average study did not adhere to 5-6 other criteria, all of which are crucial to the accurate summary and compilation of evidence in breast augmentation. The implications vary based on which criteria are not adhered to. For example, non-adherence to *Criteria 9: Appropriate methods used to combine study findings* can lead to invalid conclusions due to inappropriate pooling of study findings, while non-adherence to *Criteria 3: Comprehensive literature search* leads to suboptimal recommendations as the literature is not completely captured. As such, the moderate score is a direct reflection of the improperly designed and executed systematic reviews in breast augmentation, indicating a necessity for higher quality reviews around the topic. Additionally, there is heterogeneity within studies found in this field, with scores ranging from 2 to 8 (Figure 4). This heterogeneity shows that despite the average score being of moderate quality, there are still low methodological quality studies published within the field that can lead to misguided advice for clinicians, resulting in poor clinical care for patients undergoing breast augmentation surgeries. Comparing our mean AMSTAR score with the median AMSTAR score of 4-5 reported by Samargandi et al, systematic reviews in breast augmentation are similar in methodological quality to the other reviews in plastic surgery.^34^ However, the low frequency of reviews on the procedure is concerning, with only 22 reviews focused on breast augmentation published over the past 20 years.

From our analysis of individual AMSTAR criteria, we found that most studies adhere to *Criteria 2: Duplicate study selection and data extraction* (n = 15, 68%). This is a strength of the systematic reviews and meta-analyses included in the present study, as multiple reviewers can reduce the risk of bias and the chance for error.^37^ In contrast, few studies followed *Criteria 1: An “a priori” design was provided* (n = 6, 27%), demonstrating no evidence of a pre-established protocol or research question prior to conducting the review. This means that studies may be conducting post hoc analyses, thereby weakening the conclusions drawn from their analyses. Thus, we recommend future studies to follow an a priori design by publishing systematic review/meta-analysis protocols or registering the protocol through PROSPERO prior to conducting the study for improved transparency.

Most studies performed a comprehensive literature search (n = 17, 77%), thus adhering to *Criteria 3*. This is necessary as systematic reviews require a thorough search strategy that involves multiple databases in order to capture the scope of a specified topic.^38^ However, only 1 study (4.5%) included adhered to *Criteria 4: Status of publication used as inclusion criteria*, while only 3 studies (14%) adhered to *Criteria 10: Likelihood of publication bias assessed*. The lack of representation of gray literature in the search strategy decreases the scope of the study and the lack of publication bias assessment may overestimate the effect size of interventions.^39^ The scope and reporting of findings of systematic reviews in breast augmentation can be bolstered through the improvement of these 2 criteria.

We found that most studies assessed the scientific quality of their included studies (n = 14, 64%), in accordance with *Criteria 7*, though far fewer (n = 7, 32%) adhered to *Criteria 8: Scientific quality of included studies used appropriately in formulating conclusions*. This is an interesting finding because most studies considered that the quality of the included studies may have an effect on their conclusions as they decided to conduct the quality assessment initially but neglected to take the quality assessment into consideration when drawing conclusions. Thus, it is important to mention the quality of systematic reviews used when discussing conclusions so readers are aware that the conclusions may not be of the highest quality when considering applicability to clinical practice.

Approximately 36% of our included studies were identified from *Plastic and Reconstructive Surgery*. This is not surprising as the journal has the highest impact factor among plastic and reconstructive surgery journals and promotes the application of evidence-based medicine to clinical practice.^34^ However, both the *Aesthetic Surgery Journal* and *Aesthetic Plastic Surgery* had the second most studies identified, with 23% in each. Therefore, several journals can be explored by surgeons seeking evidence-based studies to guide breast augmentation procedures.

Analysis of country affiliation found that studies were primarily published by authors in the United States (n = 8), followed by authors from China (n = 4). This is interesting to note, as the methodological quality of articles assessed in this present study pertains specifically to the aforementioned regions. As such, we should remain cautious when interpreting studies from other regions due to the possibility of differences in healthcare systems and breast augmentation practices.

There was no significant correlation identified between AMSTAR scores and journal impact factor. This suggests that leading journals in plastic surgery may not evaluate methodological quality more than others. However, it should be noted that most studies were identified across only 3 journals. Similarly, there was no significant correlation between AMSTAR scores and number of citations, which suggests that influential studies do not necessarily have greater methodological rigor. It is surprising to see that the number of included studies is not correlated with the AMSTAR score because it is difficult to conduct statistical analyses and draw meaningful conclusions with fewer data points. However, this finding makes it clear that conducting a robust systematic review/meta-analysis involves improving the methodological quality through adherence to AMSTAR guidelines, not simply just the inclusion of more studies for analysis.

Articles that identified adherence to PRISMA guidelines were found to have higher AMSTAR scores on average as compared with those that did not (*P* = 0.03). This is in line with a previous study by Panic et al that found the endorsement of PRISMA guidelines led to increased methodological quality as measured by AMSTAR.^40^ This finding is not surprising since many of the AMSTAR criteria overlap with the specific items of the PRISMA statement. However, we must remain cognizant of the fact that self-reported adherence to PRISMA guidelines is not synonymous with actual adherence.

Previously, Samargandi et al presented a 6-step process in conducting systematic reviews based on AMSTAR criteria.^34^ However, it may be difficult for authors to conceptualize the sixth step of “Report appropriately” without consideration of how to report each step of the study. Given that PRISMA adherence was found to be correlated with AMSTAR quality, we present a revised 5-step process by integrating necessary reporting of each step according to the PRISMA statement (Table 5).

**Table 5.** Revised 5-Step Approach on How Systematic Reviews Should Be Performed Based on AMSTAR and Reported Based on PRISMA

Description of step	Conducting of SR based on AMSTAR criteria	Reporting of SR based on PRISMA
(1) Specify the clinical question and review method	*Item 1*: Use a precondition in the design of systematic reviews and define relevant populations, interventions, outcomes, study designs, search process, study selection criteria, and methods of quality assessment a priori	In the a priori protocol and final manuscript, the title should specify whether the study is a systematic review, meta-analysis, or both. The rationale, objectives, parameters collected, and analyses conducted (for both data synthesis and quality assessment) should be predefined and reported in the protocol. The final manuscript should indicate where the protocol is published and provide the web address and registration information to improve transparency and prevent post hoc analyses
(2) Identify relevant studies	*Item 2*: Extensively search in 2 or more electronic databases (eg, MEDLINE, EMBASE, and CENTRAL)	The study design should be clearly reported, including the eligibility criteria for screening, databases accessed, search strategy, and data extraction methods. When detailing the screening process, the number of studies assessed and the reason for exclusion at each stage should be specified, ideally through a flow diagram for easier access
	*Item 3*: Involve 2 or more independent data extractors and specify a consensus procedure for any discrepancies	
	*Item 4*: Consider searching for relevant unpublished works and languages other than English whenever possible	
	*Item 5*: List both included and excluded studies in tables and provides reasons for exclusion	
(3) Summarize the included studies and evaluate the quality of included studies	*Item 6*: Summarize the characteristics and relevant data of included studies and present them in a table	In the results sections, authors are recommended to include the characteristics, quality appraisal, and a simple summary of the data from each individual study included. Ensure citations are included, so readers can access the original study for reference
	*Item 7*: Assess the methodologic quality and tendency of bias of included studies. Use a previously validated appraisal tool based on the study design. Present the results of the assessments in a table	
(4) Synthesize the data, assess combinability, and summarize the evidence	*Item 9*: Use appropriate statistical methods for evaluating heterogeneity between included studies and combining their effects. Appropriate meta-analysis produces the most powerful results. If study outcome cannot be pooled, they should be explicitly summarized and presented in a table in parallel to methodologic rigor of the study	Present the findings of all analyses, including appropriate statistical measures (eg, CIs, *P* values, measures of inconsistency). Any additional analyses should be reported in the results section and specified whether they were predefined. Present any quality assessments where appropriate
(5) Interpret the findings of the review	*Item 8*: Consider the results of the scientific quality in the analysis and formulating conclusions and recommendations. Any recommendations should be graded based on the level of the quality of evidence. The higher the quality of studies, the more valid and trustable the result of SRs	When presenting the interpretation of the findings, it is important to consider and discuss the quality of evidence for each outcome. For lower quality evidence, this should be highlighted as a limitation, while also discussing potential limitations of the study design. The conclusion should provide a general interpretation of the key findings and provide implications or next steps for future research. Researchers should remember to include any sources of funding received and the role of the funders in the study to prevent any misunderstandings or inaccurate assumptions
	*Item 10*: Explore the risk of publication bias	
	*Item 11*: Acknowledge conflicts of interest of both the systematic review itself and included studies	

AMSTAR, a measurement tool to assess systematic reviews; PRISMA, Preferred Reporting Items for Systematic Reviews and Meta-Analyses; SR, systematic review.

Our synthesis of study findings revealed high patient satisfaction and low complication rates (10%-20%) with breast augmentation surgeries, regardless of surgical type (implant-based, autologous fat grafting, and augmentation-mastopexy) and technique (incision type, implant placement, etc.). Some notable conclusions reported by multiple studies include lower rates of breast cancer in implant-based augmentation compared with the general population, lower capsular contracture rates in textured implants compared with smooth implants, and a lasting improvement in breast size and shape with autologous fat grafting (Table 4). These findings are often relied upon by plastic surgeons to enhance their clinical care, who may not recognize the bias that is introduced with improperly conducted systematic reviews that renders their conclusions mute. For example, in our analysis, Stanley et al scored a 2/11, one of the lowest scores among the included studies.^27^ They concluded that the best method of non-narcotic pain relief involves breast pocket irrigation with bupivacaine and ketorolac due to its simplicity, efficacy, and intraoperative administration that removes responsibility from the patient.^27^ However, they did not conduct a comprehensive literature search (*Criteria 3*) or include gray literature (*Criteria 4*) in the execution of their study. In only searching MEDLINE and omitting a search of gray literature databases, such as SIGLE, they likely did not capture all the available literature surrounding pain control post-augmentation surgery, and there may be other options that were not considered in their analysis. Without a rigorous study design, clinicians reading this study may inappropriately recommend a suboptimal pain control method to patients undergoing breast augmentation surgery.

### Limitations

An acknowledged limitation of this study is that studies were selected that focused solely on breast augmentation. This narrowed the scope as it excluded studies with breast augmentation procedures as a specific subgroup, such as studies that assess silicone implants being used in both breast augmentation and breast reconstruction procedures.^41^ This resulted in a relatively small sample size (n = 22). Further studies in this field may reveal pertinent trends in methodological quality and study characteristics. Another limitation identified was the impact of publication date on number of citations. It stands to reason that recent studies would have less opportunities to be cited as compared with older studies. However, this characteristic was still analyzed to provide insight on whether influential studies contained higher methodological quality. Finally, there is potential for bias in the interpretation of results of AMSTAR criteria 9 (*Appropriate methods used to combine findings of studies*) and 10 (*Likelihood of publication bias assessed*) as both can be reported as “N/A” due to qualitative research questions and lack of pooling, respectively. Studies found “N/A” would skew the results down for these criteria even though they do not qualify based on study design. Therefore, it may be more appropriate to report these criteria by removing studies found “N/A” from the analysis.

## CONCLUSIONS

Overall, the methodological quality was found to be moderate, with no study achieving good quality. The major criteria negatively impacting AMSTAR scores included status of publication used as inclusion criteria and list of studies provided. Systematic reviews and meta-analyses that reported adherence to PRISMA guidelines were found to achieve greater AMSTAR scores, indicating higher methodological quality. The overall moderate quality is indicative of poor study design and execution, impairing the quality of findings that can be concluded by these systematic reviews. It indicates a necessity for higher quality summaries and pooling of available evidence to drive clinical decision making. Prior to publication, journal reviewers and editors should appraise systematic reviews and meta-analyses using the AMSTAR tool and ensure reporting follows the PRISMA guidelines to promote higher quality evidence in breast augmentation procedures. Similarly, when interpreting systematic reviews, we recommend clinicians to become familiar with individual AMSTAR criteria and recognize the implications of not adhering to that criteria when deciding whether a study’s recommendations should be brought to patient care. Researchers, reviewers, and clinicians should understand the consequences of a poorly designed systematic review: the conclusions derived from these studies may not be reflective of the available evidence pool and may lead to the implementation of suboptimal, ineffective, or even harmful clinical practices.

## Supplementary Material

ojab020_suppl_Supplementary_Appendix
