# Supplementary material for: Evaluating the Quality of Systematic Reviews and Meta-Analyses About Breast Augmentation Using AMSTAR
Source: Aesthet Surg J Open Forum. 2021 May 22;3(3):ojab020. doi: 10.1093/asjof/ojab020 (PMC8259036; doi:10.1093/asjof/ojab020)
Supplement: ojab020_suppl_Supplementary_Appendix [file ojab020_suppl_supplementary_appendix.docx]

**SUPPLEMENTAL MATERIAL**

MEDLINE and EMBASE

1. systematic review.mp.
2. meta-analysis.mp.
3. or/1-2
4. plastic & reconstructive surgery.jn.
5. aesthetic surgery journal.jn.
6. jama facial plastic surgery.jn.
7. burns.jn.
8. "journal of plastic reconstructive & aesthetic surgery jpras".jn.
9. "british journal of plastic surgery".jn.
10. "journal of hand surgery european volume".jn.
11. "journal of hand surgery british volume".jn.
12. "journal of hand surgery american volume".jn.
13. microsurgery.jn.
14. "journal of cranio maxillo facial surgery".jn.
15. "journal of reconstructive microsurgery".jn.
16. "journal of burn care & research".jn.
17. "journal of burn care & rehabilitation".jn.
18. "annals of plastic surgery".jn.
19. aesthetic plastic surgery.jn.
20. facial plastic surgery.jn.
21. "scandinavian journal of plastic & reconstructive surgery & hand surgery".jn.
22. clinics in plastic surgery.jn.
23. or/4-22
24. 3 and 23
25. limit 24 to yr="2000-2019"

Cochrane Library of Systematic Reviews

1. (Plastic and Reconstructive Surgery):so
2. (Aesthetic Surgery Journal):so
3. (JAMA Facial Plastic Surgery):so
4. (Burns):so
5. (Journal of Plastic Reconstructive and Aesthetic Surgery):so
6. (Journal of Hand Surgery):so
7. (Journal of Hand Surgery European Volume):so
8. (Journal of Hand Surgery American Volume):so
9. (Microsurgery):so
10. (Journal of Cranio maxillofacial Surgery):so
11. (Journal of Reconstructive Microsurgery):so
12. (Journal of Burn Care and Research):so
13. (Annals of Plastic Surgery):so
14. (Aesthetic Plastic Surgery):so
15. (Facial Plastic Surgery):so
16. (Clinics in Plastic Surgery):so
17. #1 OR #2 OR #3 OR #4 OR #5 OR #6 OR #7 OR #8 OR #9 OR #10 OR #11 OR #12 OR #13 OR #14 OR #15 OR #16
18. (systematic review):ti,ab,kw
19. (meta-analysis):ti,ak,kw
20. #18 OR #19
21. #17 AND #20
